# Synthesis and Physicochemical Properties of Acrylate Anion Based Ionic Liquids

**DOI:** 10.3390/polym14235148

**Published:** 2022-11-26

**Authors:** Veronika S. Fedotova, Maria P. Sokolova, Vitaliy K. Vorobiov, Eugene V. Sivtsov, Mauro C. C. Ribeiro, Michael A. Smirnov

**Affiliations:** 1 Institute of Macromolecular Compounds, Russian Academy of Sciences, V.O. Bolshoi Pr. 31, 199004 St. Petersburg, Russia; 2 Saint Petersburg State Institute of Technology, Moskovsky Prospekt 24-26/49, 190013 St. Petersburg, Russia; 3 Laboratório de Espectroscopia Molecular, Departamento de Química Fundamental, Instituto de Química, Universidade de São Paulo, São Paulo 05513-970, Brazil; 4 Institute of Chemistry, Saint Petersburg State University, Universitetsky Pr. 26, Peterhof, 198504 St. Petersburg, Russia

**Keywords:** bacterial cellulose nanofibers, polymerizable ionic liquids, 3D printing, rheological properties, cellulose structure

## Abstract

Two polymerizable ionic liquids (or monomeric ionic liquids, mILs) namely 1-butyl-3-methylimidazolium and choline acrylates ([C_4_mim]A and ChA, respectively) were synthesized using the modified Fukumoto method from corresponding chlorides. The chemical structure of the prepared mILs was confirmed with FTIR and NMR study. Investigation of the thermal properties with DSC demonstrates that both mILs have a *T*_g_ temperature of about 180 K and a melting point around 310 K. It was shown that the temperature dependence of FTIR confirm the *T*_g_ to be below 200. Both mILs exhibited non-Newtonian shear thinning rheological behavior at shear rates >4 s^−1^. It was shown that [C_4_mim]A is able to dissolve bacterial cellulose (BC) leading to a decrease in its degree of polymerization and recrystallisation upon regeneration with water; although in the ChA, the crystalline structure and nanofibrous morphology of BC was preserved. It was demonstrated that the thixotropic and rheological properties of cellulose dispersion in ChA at room temperature makes this system a prospective ink for 3D printing with subsequent UV-curing. The 3D printed filaments based on ChA, containing 2 wt% of BC, and 1% of N,N′-methylenebisacrylamide after radical polymerization induced with 1% 2-hydroxy-2-methylpropiophenone, demonstrated Young’s modulus 7.1 ± 1.0 MPa with 1.2 ± 0.1 MPa and 40 ± 5% of strength and ultimate elongation, respectively.

## 1. Introduction

Ionic liquids (ILs) are a unique class of compounds with a melting point below 100 °C. They are typically composed of an organic cation (the most often used include dialkylimidazolium [1], alkylpyridinium [2] or tetraalkylammonium [3]) and an organic or inorganic anion [4]. ILs demonstrate high thermal stability [5] and ionic conductivity [6], low vapor pressure [7], and electrochemical stability [6]. These properties determine interest toward them as possible solvents for liquid chromatography in the analysis of drugs [8], catalysis [9], and the extraction of metals [10], as well as the synthesis, modification and processing of macromolecular compounds [11].

In the field of polymer science and technology, growing interest has arisen in the area of ILs capable of polymerization (or monomeric ILs, that can be noted as mILs). This type of ILs ensure the possibility of solidifying (or sharp viscosity increasing) of the material under external stimuli [12,13] due to the formation of the polymer. As a result, materials with the mechanical properties of polymers, and the physicochemical properties of ILs can be formed [14]. For example, the ionic conductivity and low flammability of ILs are preserved in polymers based on them [15]. These properties make mILs and their corresponding polymers interesting for use as catalysts [16], the production of porous materials [17], the absorption of CO_2_ [18], and in a biologic application, for example, for the manufacture of biosensors [19].

UV-curable mILs contain polymerizable unsaturated groups either in the structure of a cation or anion [20]. In both cases, it is necessary to synthesize an IL monomer that will contain the carbon–carbon double bond. A common method used for the synthesis of mILs is the quaternization of heterocyclic compounds containing a vinyl group, followed by anion replacement or protonation with the corresponding acid [20,21]. In this way, mIL has been obtained by the quaternization of 1-vinylimidazole with octyl bromide [22]. The polymerization of the resulting 3-octyl-1-vinylimidazolium bromide was carried out using ultrasonic treatment in combination with the initiator 4,4′-azobis (4-cyanopentanoic acid). A methacrylate group is also used to form mILs with a polymerizable cation [23,24]. In this case, the synthesis of the monomer includes several stages. Most often, methacrylate derivatives, such as 2-bromoethyl methacrylate [25] or 2-chloroethyl methacrylate [26], are obtained by the introduction of methacryloyl chloride to a quaternization reaction with substituted imidazole.

mILs based on the polymerizable quaternary ammonium cation are also reported [27,28]. Among them, the choline containing mILs were reported as monomers with lower toxicity [29]. The corresponding monomers can be obtained from 2-dimethylaminoethyl methacrylate, which already contains a polymerizable group [27], using the sequence of quaternization and anion exchange reactions. By this method, the 2-cholinium lactate methacrylate was obtained from 2-dimethylaminoethyl methacrylate [30].

The number of examples of mILs with polymerizable anions is lower than ones with polymerizable cations. For example, mIL with a polymerizable sulfonate-based anion was obtained as a result of an anion exchange reaction between tetra-n-butylphosphonium chloride and sodium styrene sulfonate [31] or via the reaction between tributylhexylphosphonium bromide and potassium 3-sulfopropylmethacrylate [32]. In other works, mILs containing polymerizable carboxylate anions were obtained using the reaction between acrylic acid and N-ethylimidazole [33] or by a metathesis reaction between acrylic acid and choline bicarbonate [34]. However, the number of studies devoted to the obtaining of mILs with polymerizable anions remains few. To the best of our knowledge, the thermal and rheological properties of mILs containing acrylate anions have not been studied in detail previously. In the present work, the modified Fukumoto method [35] described by Korchak et al. [36] was used to prepare mILs with unsaturated polymerizable acrylate anions and the two most frequently studied cations used in ILs: 1-butyl-3-methylimidazolium and choline, for the first time. The advantages of this method are the simplicity of the mILs preparation and the possibility to start from chlorides—the most common and cheap salts of the mentioned cations. According to this method, 1-butyl-3-methylimidazolium acrylate ([C_4_mim]A) and choline acrylate (ChA) were synthesized. The structure, thermal and rheological properties of prepared mILs were also characterized.

The possibility of ILs and their new analogs—deep eutectic solvents (DES)—to be solvents or dispersive media for natural polymers is often emphasized. This property is used for polysaccharide processing [37,38], for the preparation of self-healable ion conducting hydrogels [39,40,41], and in the 3D printing of sensors [42]. Cellulose, as the most abundant polymer, is often used in such materials [43]. In the context of the mentioned application, the phase transition temperatures and rheological properties of mILs and their compositions with cellulose is of prime importance. The dependence upon viscosity, shear rate and thixotropic behavior are the main parameters that determine the applicability of inks for 3D printing with high shape fidelity [44,45]. Recently, it was demonstrated that the appearance of an –OH group can change the local dynamics of ILs by several orders of magnitude and its influence is dominant in comparison with Coulombic interactions [46]. Thus, the higher viscosity and yield strength of ChA containing an –OH group over a [C_4_mim]A without this group can be expected to be significant for its application in 3D printing. This point has not been studied yet. Accordingly, the rheological properties of mILs/cellulose dispersions and their 3D printability was investigated in this work. For a clearer interpretation of the results, the structure of the cellulose, regenerated from mILs, was characterized by XRD, AFM and molecular mass measurements. The cellulose of bacterial origin (bacterial cellulose—BC) was chosen for this study because of its high purity, as well as its well characterized and stable crystalline structure and morphology. 

## 2. Materials and Methods

### 2.1. Materials

Choline chloride, ChCl (CAS 67-48-1, purity > 99%) was purchased from Glentham Life Sciences Ltd. (Corsham, UK). Residual water was removed in a vacuum at 60 °C for 24 h. 1-butyl-3-methylimidazolium chloride, [C_4_mim]Cl (CAS 79917-90-1, purity > 98%, Buchs SG, Switzerland), acrylic acid (CAS 607-061-00-8, purity > 99%, Prague, Czech Republic), N,N′-methylene-bis-acrylamide (CAS 110-26-9, purity > 99.5%, St. Louis, MO, USA) and 2-hydroxy-2-methylpropiophenone (CAS 7473-98-5, purity > 97%, Milan, Italy) were obtained from Sigma-Aldrich and used as received. KOH (purity > 99%) and methanol (purity > 99.5%) were purchased from VEKTON (Saint Petersburg, Russia). BC was obtained with *Acetobacter xylinum*, which was purchased from the All-Russian collection of industrial microorganisms (National Bioresource Center, GosNIIgenetika, Moscow, Russia). Peptone and D-mannitol (CAS 69-65-8) were obtained from LenReaktiv (Saint Petersburg, Russia). Yeast extract for preparation of the culture medium was received from the Research Center for Pharmacotherapy (Saint Petersburg, Russia). The obtained BC was purified using NaOH (CAS 1310-73-2) from NevaReaktiv (Saint Petersburg, Russia).

### 2.2. Methods

#### 2.2.1. Synthesis of mILs

**Scheme 1:**
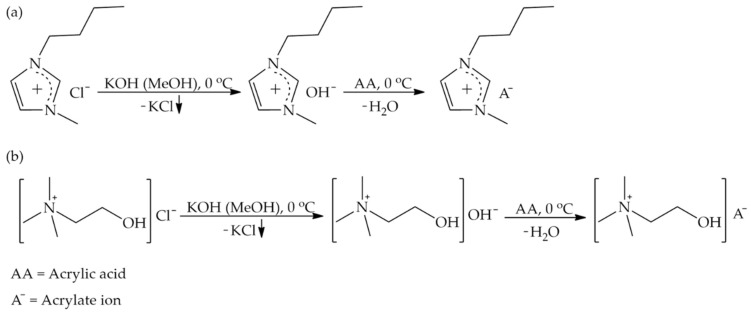
Synthesis scheme of [C_4_mim]A (**a**) and ChA (**b**).

mILs containing acrylate ions were synthesized by a procedure based on the modified Fukumoto method [35], which has been described by Korchak et al. [36]. This method consists of a two-stage process (Scheme 1). The synthesis was carried out in a thermostatically controlled cell at 0 °C. In the first stage, 1-butyl-3-methylimidazolium hydroxide ([C_4_mim]OH) or choline hydroxide (ChOH) were obtained as a result of an exchange reaction between 15.034 g of [C_4_mim][Cl] or 15.044 g of ChCl and a 30 wt% KOH solution in methanol. As a by-product, KCl was precipitated and removed by filtration. In the second stage, the solution of [C_4_mim]OH or ChOH was added drop by drop to 6.825 g or 8.541 g of acrylic acid (AA) (taken in a small excess). A neutralization reaction was conducted at 0 °C. The content of the residual chlorides in the prepared liquids was estimated using the gravimetric method by careful collection, drying and weighing of the KCl obtained at the first stage. It was found that, in the case of [C_4_mim]A, the yield of KCl was 99.8 wt%, while in the case of ChA, the yield was 97.5 wt%. Accordingly, the content of the residual unreacted chloride salt can be estimated as 0.19 and 2 wt% for [C_4_mim]A and ChA, respectively. Further, methanol was distilled from the reaction mixture using a rotary evaporator at 40 °C and reduced pressure. After the removal of the methanol, the temperature was increased to 70 °C to remove the excess AA. As a result, white gel-like (at room temperature) mILs were obtained. In the text, they will be denoted as [C_4_mim]A (1-butyl-3-methylimidazolium acrylate) and ChA (choline acrylate).

#### 2.2.2. Dissolution and Regeneration of Cellulose

BC was synthesized according to the method described in [47]. For this purpose, the lyophilized *A. xylinum* was activated and then cultured using a culture medium. An obtained cell suspension was introduced into the additional portion of the culture medium, and was statically incubated in an incubator (BINDER, Tuttlingen, Germany) at 27 °C for a duration of 15–20 days. The resulting BC membrane was collected from the top of the medium and purified with an NaOH solution and washed with distilled water until pH 7 was reached. The purified BC gel membrane was then mechanically crushed with a blender, diluted with water, and then lyophilized using a Scientz-10ND freeze dryer (Scientz, Ningbo, China). For the preparation of the mILs/cellulose dispersion, the portions of 178 and 224 mg ground freeze-dried BC were mixed with 8.746 and 10.976 g of ChA and [C_4_mim]A, respectively, in order to obtain dispersions with 2 wt% of cellulose content. The obtained viscous masses were stirred for 3 days at 60 °C until the cellulose was completely wetted with solvent. Then, the dispersions were homogenized by repeatedly passing them through nozzles with a 0.41 mm diameter. The cellulose was regenerated from BC/ChA and BC/[C4mim]A by a treatment of dispersion with excess of water to characterize its structure. Then cellulose was separated with centrifugation using a centrifuge (Digisystem Laboratory Instruments Inc., New Taipei City, Taiwan) at 4000 rpm for 20 min. Removal of the residual mILs was achieved by repeating the washing of the cellulose with distilled water and centrifugation 7 times. Then, the cellulose was air dried. The degree of polymerization (DP) of the initial BC and the BC after regeneration from the BC/[C_4_mim]A and BC/ChA dispersion was determined by the characteristic viscosity [η]. The method based on cellulose dissolution in an iron (III) sodium tartrate complex was applied. The DP was calculated according to the equation [η] = 2.74 × 10^−2^ DP^0.775^ [48].

#### 2.2.3. Structural Study of mILs

The chemical structure of the synthesized mILs was confirmed by ^1^H and ^13^C NMR spectroscopy and infrared spectroscopy with Fourier transform (FTIR). The NMR Fourier spectrometer AVANCE II-500WB (Bruker, Billerica, MA, USA) was used. D_2_O was used as a solvent. FTIR spectra were measured in transition mode for the mILs distributed between crystal plates of thallium bromide (KRS-5) using the IRAffinity-1S spectrometer (Shimadzu, Kyoto, Japan). Additionally, the IR spectra of [C_4_mim]A and ChA at different temperatures were obtained in transmission mode in a FTIR Bruker spectrometer model Vertex 80v (Ettlingen, Germany) as a function of temperature (190 K < *T* < 340 K) using the Linkam temperature control model FTIR6000 with ZnSe windows on the stage of a Hyperion microscope coupled to the spectrometer. The sample within the temperature control was placed on a KRS-5 disc, and the sample chamber was under a continuous flow of nitrogen. The temperature was decreased from room temperature to 190 K at a rate of −5 K/min, and then stepwise increased to 330 K. The spectra were acquired with a spectral resolution of 4.0 cm^−1^, and 32 scans were accumulated at each temperature. The IR spectra of [C_4_mim]Cl and ChCl were obtained using KBr. The obtained mILs were also characterized by mass spectrometry using a triple quadrupole mass spectrometer LCMS-8040 (Shimadzu, Kyoto, Japan) in negative electrospray ionization mode.

#### 2.2.4. Wide-Angle X-ray Diffraction Study

The crystalline structure of the regenerated BC was studied by Wide-angle X-ray diffraction (WAXD) with a Rigaku SmartLab 3 diffractometer (Rigaku Corporation, Tokyo, Japan) equipped with a CuK_α_ radiation source (λ = 1.54 Å) within the 2θ range of 5°–40° with the scan step of 0.05°.

#### 2.2.5. Microscopic Investigation

Atomic force microscopy (AFM) studies were performed using the SPM-9700HT scanning probe microscope (Shimadzu, Kyoto, Japan), and 512 × 512 pixel images were obtained in the tapping mode with a NSG30SS silicon tip (curvature radius 2 nm). Experimental data were processed using the SPM software v.4.76.1 (Shimadzu, Kyoto, Japan) software. 

#### 2.2.6. Investigation of Thermal Properties of mILs

A thermogravimetric analysis (TGA) of the synthesized mILs was performed on a Shimadzu DTG-60 Plus instrument (Kyoto, Japan) in an argon atmosphere with samples of approximately 15 mg at a scanning speed of 10 °C/min from 298 K to 973 K. Data processing was carried out using the ShimadzuCorporation©ta60 Version 2.21. 

Differential scanning calorimetry (DSC) was performed using a Shimadzu DSC-60 Plus differential scanning calorimeter (Kyoto, Japan). The analysis was carried out in air with samples of approximately 5.1–5.3 mg at a scanning speed of 10 °C/min in the temperature range from 123 K to 373 K.

#### 2.2.7. Rheological Studies

Rheological measurements of ChA, [C_4_mim]A and BC/ChA, BC/[C_4_mim]A were performed using a Physica MCR 302 rheometer (Anton Paar, Graz, Austria) with the plane–plane measuring system having a diameter of 25 mm and a gap of 0.4 mm. Data processing was performed using the software RHEOPLUS/32 V.3.62 (Anton Paar, Ostfildern, Germany). In the case of the pure mILs, steady shear measurements were carried out with an increase in the shear rate in the range 1–100 s^−1^ at 298 K. 

Steady shear measurements of BC/ChA and BC/[C_4_mim]A were carried out with an increase in the shear rate in the range of 1–100 s^−1^ at 298 K. The dynamic rheological behavior was studied at 298 K. The limit of the linear viscoelasticity (LVE) region was determined by testing for the sweep of the deformation amplitude at a frequency of 1 Hz in the upward sweep mode. It was found that the values of the limit of the LVE region were 0.1 and 0.4% for BC/ChA and BC/[C_4_mim]A, respectively (Electronic Supplementary Information (ESI), Figure S1a). This result was used to perform an angular frequency sweep in the LVE regime using the frequency range from 0.1 to 100 rad∙s^–1^. The three-interval thixotropy tests were performed via a measurement of apparent viscosity at a shear rate of 0.01 s^–1^ for 45 s followed by a sharp increase of up to 3000 s^–1^ for 1 s and then a decrease again to 0.01 s^–1^. The viscosity at the last step was measured for at least 500 s to observe the recovery of the structure.

#### 2.2.8. Application of ChA and [C_4_mim]A for 3D Printing with BC

The UV-curable compositions for 3D printing were prepared by adding to the 2.526 g of BC/ChA and 2.013 g of BC/[C_4_mim]A dispersions, respectively: 10 mg and 7 mg of a photo initiator (2-hydroxy-2-methylpropiophenone); and 10 mg and 7 mg of a bifunctional crosslinking agent (N,N′-methylene-bis-acrylamide). The content of the photo initiator and the crosslinking agent were 1 wt% for both, respectively, to the mass of the polymerized monomer (acrylate ion). Before printing, the samples were centrifuged at 4000 rpm for 20 min to remove air bubbles. The obtained compositions were used for the 3D printing of strips in order to obtain data on the mechanical characteristics of filaments. Printing and UV-curing were performed using a 3D BioScaffolder BS3.2 (Gasim, Radeberg, Saxony, Germany), equipped with a pneumatic syringe (nozzle with a diameter of 0.58 mm) and UV-lamp OmniCure S1500 (Lumen Dynamics, Mississauga, ON, Canada) operating at light power 2 W∙cm^−2^.

The mechanical measurements of printed strips were carried out in the tensile mode at a traverse speed of 10 mm∙min^–1^ on an Instron 5943 universal testing instrument (Instron, Norwood, MA, USA). The test strips have the gauge length of 2 cm. Prior to testing, the samples were kept for 3 days in a desiccator with CaCl_2_. The cross section of each stripe was measured using an Altami 104 (Altami, Saint Petersburg, Russia) equipped with a 1.3 MP digital camera VEC-135 (EVS, Saint Petersburg, Russia) by processing the photos within ImageJ software v.2.0.0 (National Institutes of Health, Bethesda, MD, USA. A statistical analysis was performed based on the measurements of 7 samples.

## 3. Results and Discussion

### 3.1. Synthesis and Spectroscopic Study of Acrylate-Based mILs

**Figure 1:**
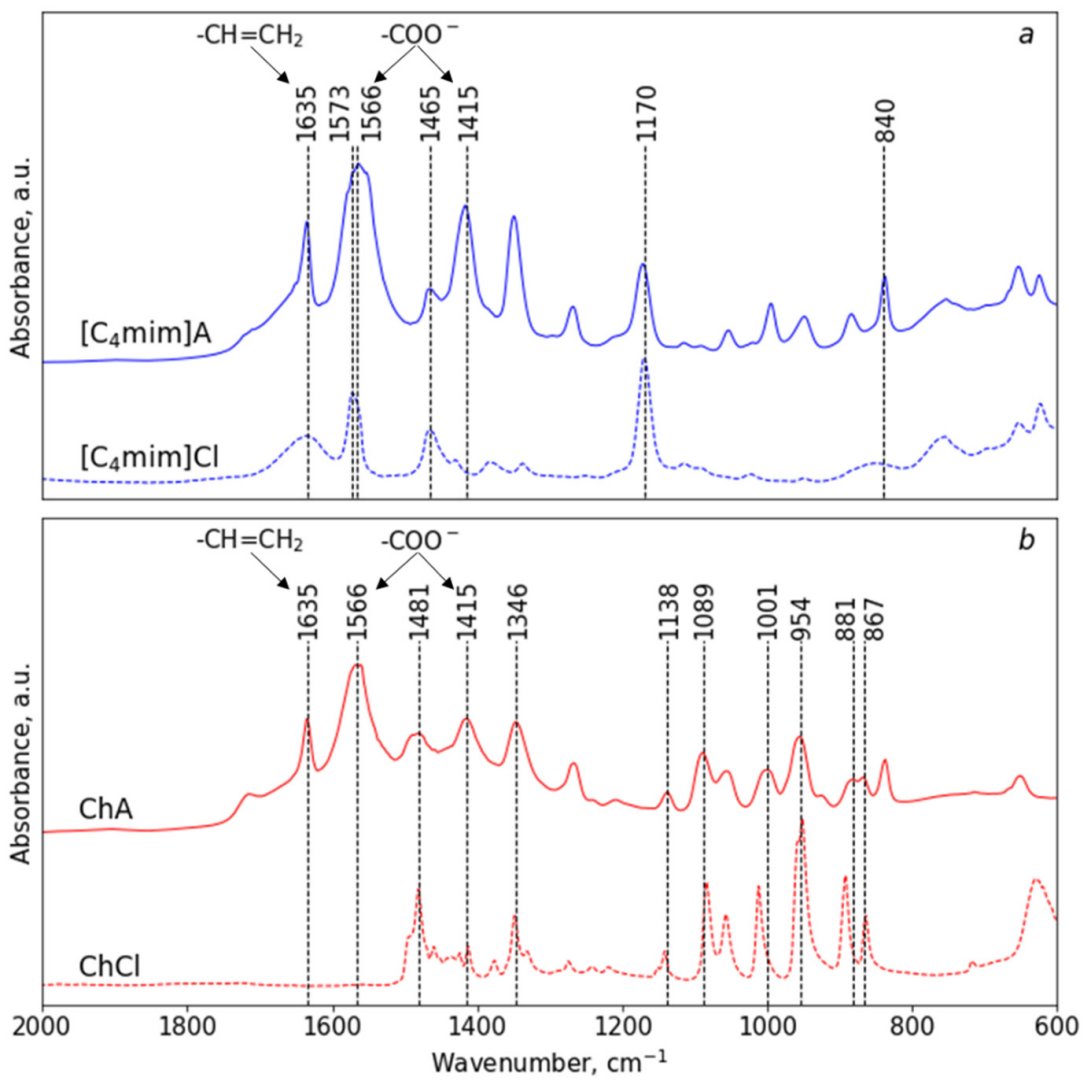
FTIR spectra of the starting imidazolium chloride and resulting [C_4_mim]A (**a**); starting choline chloride, and resulting ChA (**b**).

The results of the ^1^H and ^13^C NMR spectroscopy confirmed the structure of the obtained mILs based on acrylate ions; the spectra are presented in ESI (Figure S2a,b and Figure S3a,b, respectively). Figure 1a,b shows the FTIR spectra of the initial chloride salts and corresponding mILs. Typical peaks for the 1-butyl-3-methylimidazolium cation that are visible on the spectrum of [C_4_mim]Cl are also observed in the spectrum of [C_4_mim]A (Figure 1a) [49]. In the case of [C_4_mim]A, the bands at wave numbers 1635 and 1573 cm^−1^ are associated with the stretching vibrations of the C=C and C=N bonds in the imidazolium ring, respectively. The peak, which is observed at 1465 cm^−1^, is due to the asymmetric bending of the CH_3_ and CH_2_ groups of alkyl substituent of the 1-butyl-3-methylimidazolium cation. The peak at 1170 cm^−1^ is connected to the stretching of the C–N bond. The peak at the wavenumber near 840 cm^−1^ is due to the C–N stretching vibration [50,51].

Peaks near 1481, 1346, 1138, 1089, 1001, 954, 881, 867 cm^−1^, typical of the choline cation and clearly visible on the spectrum of ChCl [52], are also observed on the spectrum of ChA (Figure 1b). 

In the spectra of both mILs bands near 1635 cm^−1^ (–CH=CH_2_ double bond vibration) and 1566, 1415 cm^−1^ (asymmetric and symmetric vibrations of carboxylate group, respectively) are appearing in comparison with the starting chlorides. This demonstrates the successful conversion of the chlorides to acrylates. It is worth noting that in the case of [C_4_mim]A, the peak of the asymmetric vibration carboxylate groups near 1566 cm^−1^ is overlapped with the peak of the imidazolium ring.

**Figure 2:**
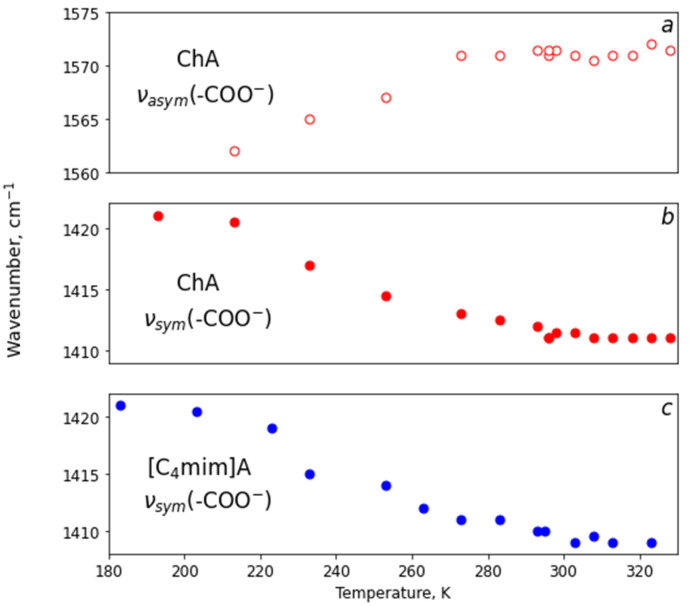
Dependence of the maximums of peaks corresponding with the asymmetrical and symmetrical vibrations of the carboxylate group in ChA (**a**,**b**) and [C_4_mim]A (**c**) on temperature.

In order to study the variation in the mILs’ structure on temperature and to verify the data of the DSC study discussed, further measurements of FTIR spectra were performed in the temperature range from 190 to 340 K. Significant shifting of the position of bands connected with the symmetrical and asymmetrical vibrations of carboxylate groups was observed. In the case of ChA, both bands are clearly distinguishable because they do not overlap with the choline bands. The variation in positions of the peak maximums for ChA are given in Figure 2a,b; although in the case of [C_4_mim]A, the band centered near 1566 cm^−1^ (asymmetric vibrations of –COO^−^ group) overlap with the imidazolium ring vibration modes (Figure 1a) [53] and the position of its maximum is difficult to extract. Thus, only the variation of the peak corresponding with the symmetric vibration of the –COO^−^ group is given for [C_4_mim]A (Figure 2c). It is seen, in Figure 2, that the maximum symmetric vibration of the carboxylate groups shows a red shift, with the maximal slope between 200 and 300 K. Additionally, the plateaus at *T* < 200 K and *T* > 300 K are observed. The position of the maximum for the asymmetrical vibration band (1560–1572 cm^−1^) demonstrates a blue shift. The positions of these bands are highly sensitive to the chemical surroundings of the carboxylate group due to ion–ion interactions; for instance, complexation with cations [54]. Thus, it can be proposed that the most significant changes take place between 200 and 300 K and this can be attributed to the transformation of the structure of the mILs between *T*_g_ and *T*_m_. This result is in good agreement with the DSC data that will be discussed further. 

The increasing difference between the maximums for asymmetrical and symmetrical bands: $\Delta =\nu_{as}\left(COO\right)-\nu_{s}\left(COO\right)$ at heating can be connected with the changing of carboxylate coordination from bidentate to monodentate [53]. This process starts at 200 K and finishes at about 300 K. That can be explained by a gradual decreasing of order in the molecular structure of prepared acrylate-based mILs in a given temperature range.

### 3.2. Thermal Properties of Acrylate Containing mILs

**Figure 3:**
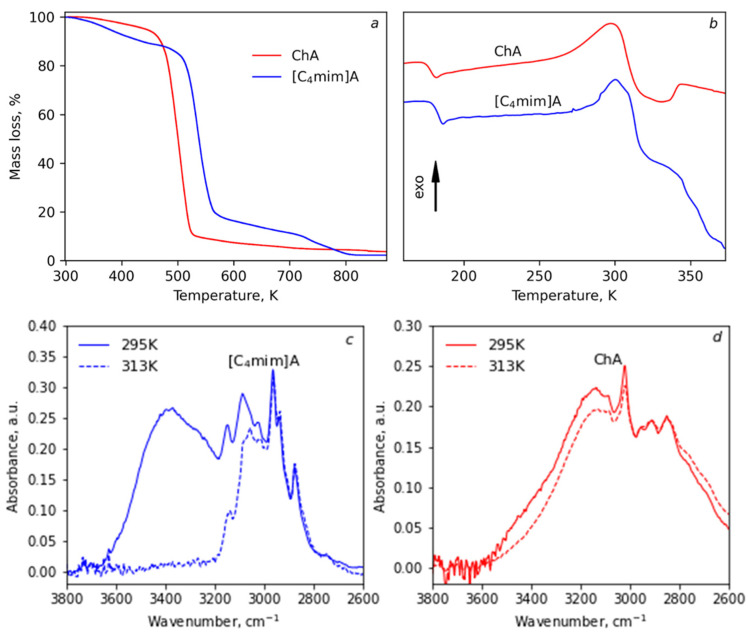
TGA curves (**a**), DSC curves (**b**), and FTIR spectra of [C_4_mim]A (**c**) and ChA (**d**).

Thermal stability and the temperatures of phase transitions in mILs are essential parameters when determining their possible practical application. In this work, the thermal properties were determined from the TGA and DSC experiments (Figure 3a,b, respectively).

The TGA curves (Figure 3a) indicate that the first region of weight loss took place at the temperature range 323–433 K. A visible 5–10% of mass loss in this region can be connected to the evaporation of water. It can be seen that the weight loss during this step was more pronounced for [C_4_mim]A. The next step on the TGA curves (Figure 3a) corresponds to the thermal decomposition of the mILs. The temperatures relating to 5% mass loss for the mILs during this process (τ_5_) were 470 K and 508 K for ChA and [C_4_mim]A, respectively, demonstrating the higher thermal stability of imidazolium based mIL. The phase transition temperatures for prepared mILs were determined from the DSC curves shown in Figure 3b. It is clearly seen that the mILs have glass transition temperatures at 178 K and 182 K for ChA and [C_4_mim]A, respectively. In addition, mIL also display cold crystallization at 298 K (ChA) and 300 K ([C_4_mim]A), followed by a broad melting transition at ca. 327 K (for both mILs). In the case of [C_4_mim]A, the baseline slope is observed on the DSC curve in the region of the melting transition. It was suggested that this slope can be connected with the evaporation of water at temperatures >310 K. To check this, the FTIR spectra for both mILs were measured at 295 and 313 K (see Figure 3c,d). The peak connected with the vibrations of bonded water centered near 3400 cm^−1^ has completely disappeared in the spectra of [C_4_mim]A after heating (see Figure 3c). In the IR spectrum of ChA at 295 K, the H_2_O band at 3400 cm^−1^ (full line in Figure 3d) is seen as a bump on the high-frequency side of the –OH band of the choline centered near 3130 cm^−1^. Water elimination upon the heating of ChA results in a decrease in the intensity of this shoulder (dashed line in Figure 3d). By comparing the spectra of both mILs in the region of the water vibrations, it can be proposed that the changes are more pronounced for [C_4_mim]A. It can be proposed that in the case of [C_4_mim]A, the evaporation of absorbed water starts at lower temperatures in comparison with ChA, which is connected with the higher polarity of the choline ion in comparison with the imidazolium one. Thus, the baseline slope on the DSC curve for [C_4_mim]A at temperatures >310 K can be attributed to the exothermic effect of water evaporation. This effect overlaps with the peak corresponding to the melting of [C_4_mim]A, that appears as a shoulder in the temperature range 300–320 K.

### 3.3. Rheological Properties of Acrylate-Based mILs

**Figure 4:**
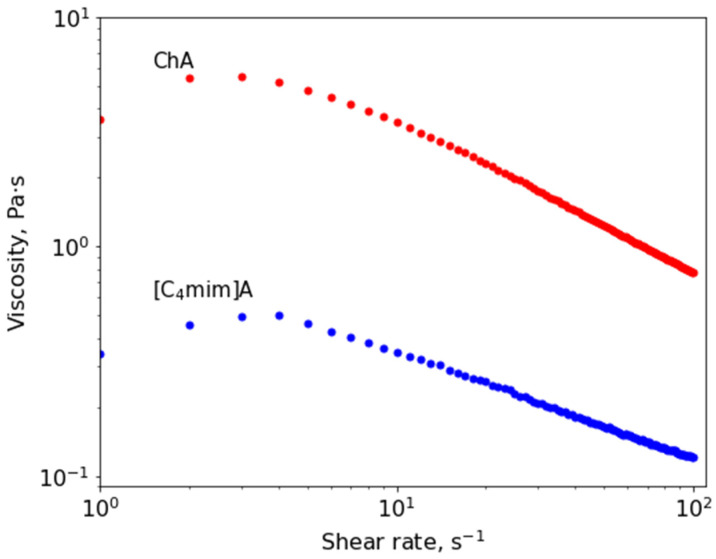
Dependence of the viscosity of pure mILs on the shear rate at *T* = 298 K.

Figure 4 shows the viscosity curves of pure mILs. For ChA, the viscosity values are an order of magnitude higher than the viscosity of [C_4_mim]A. This may be due to the presence of an –OH group in the structure of the choline cation, which leads to hydrogen bonding. It is noted in the literature that the introduction of an –OH group into the structure of an ionic liquid leads to an increase in viscosity. For example, there is an increase in viscosity at room temperature from 32.5 mPa∙s up to 85.5 mPa∙s and the introduction of –OH groups was also reported for [C_2_mim][NTf_2_] and [C_2_OHmim][NTf_2_], respectively [55].

In addition, for both mILs, the viscosity increases (at shear rates <4 s^−1^), and then a shear thinning behavior is observed. The increase in viscosity at the initial shear rates may be associated with structure formation, in particular, with the crystallization process. For example, shear thickening behavior was observed earlier for the dispersions of silica nanoparticles in imidazolium-based ILs containing BF_4_-anion and [C_2_OHmim][NTf_2_] [55]. The possible explanation of shear thickening via friction forces between the particles of dispersion was suggested only recently [56,57]. The DSC results discussed earlier demonstrate that the *T*_m_ of prepared mILs is close to the room temperature. Recent rheology and Raman spectroscopy study of the closely related system choline acetate, ChAc, showed that along with the process of melting at ~335 K, which is seen as a broad band covering ~30 K of temperature range in the DSC curve, partial recrystallization with concomitant stiffening of the sample also takes place [57]. Thus, it can be proposed that, at room temperature, some amount of crystallized mILs particles are existing in the studied objects that can lead to the shear thickening behavior of [C_4_mim]A and ChA prepared in this work. Additionally, the shear thickening behavior can be connected with the ionic packing and orientation in the bulk fluid during the shearing action [58]. The shear thinning behavior of mILs at a shear rate >4 s^−1^ can be connected with the disruption of intermolecular bonds in the ILs. 

### 3.4. Application of ChA and [C_4_mim]A for Cellulose Treatment

ILs are usually applied for the processing [59] and dissolution [60] of natural polysaccharides, especially cellulose, that is hardly dissolved in regular solvents. Since ILs can intensively interact with polysaccharides leading to their hydrolysis [61,62], the significant point to study in this field is the influence of ILs’ treatment on the structure and degree of polymerization (DP) of cellulose. The DP of initial and regenerated samples were studied with viscometry and the results are given in Table 1. It is shown that the dissolution of BC in [C_4_mim]A leads to the depolymerization of cellulose: the DP is reduced by almost two times. This is in agreement with literature data for imidazolium-based ILs. For example, the authors of the work [63] showed that the dissolution of cotton lint in 1-allyl-3-methylimidazolium chloride for 6.5 h leads to a decrease in the DP from 3133 to 2680. The use of 1-ethyl-3-methylimidazolium acetate [64] and 1-ethyl-3-methylimidazolium diethyl phosphate as a cellulose solvent also leads to its depolymerization. Zhou et al. found that during the dissolving of cellulose in 1-ethyl-3-methylimidazolium diethyl phosphate for 72 h at 130 °C, the DP is reduced by more than half [65]. At the same time, as it is seen from Table 1 in the case of BC after ChA, the DP changes insignificantly.

### 3.5. Structure and Morphology of BC after Regeneration from mILs

**Figure 5:**
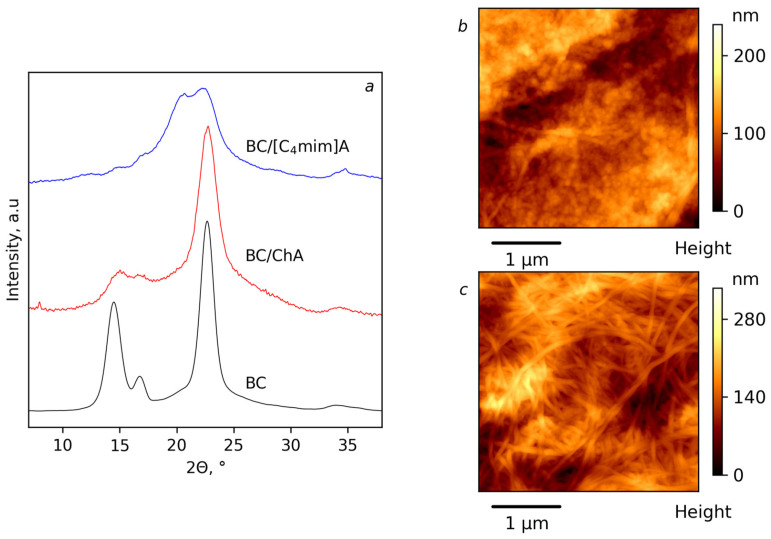
X-ray diffraction patterns obtained from BC samples (**a**), AFM images of BC/[C_4_mim]A (**b**), and BC/ChA (**c**).

The structure details of the samples were investigated with wide angle X-ray diffraction (Figure 5a). In the WAXD pattern of the initial BC (Figure 5a), three main characteristic peaks centered at 2θ = 14.5°, 16.7° and 22.7° are observed. Cellulose produced by bacteria is enriched with I*_α_* allomorph [66,67] and visible peaks can be assigned to the (100), (010) and (110) crystallographic planes of the triclinic I*_α_* allomorph [68,69,70]. The BC regenerated after treatment with ChA displayed one apparent strong diffraction peak at 2θ of 22.7° and weak peaks at 2θ of 14.5° and 16.7° (Figure 5a, BC/ChA). This indicates the presence of type I crystalline cellulose. However, a decrease in intensity and a broadening of peaks indicate a decrease in the degree of crystallinity, and/or a decrease in crystallite size [71]. The WAXD pattern of regenerated BC after [C_4_mim]A treatment are given in Figure 5a (BC/[C_4_mim]A). For this sample, the main peak at 22.7° disappeared, a broad asymmetric peak consisting of a doublet at 20.0 and 21.7° appeared, and a new peak emerged at ~12.1°. These changes indicate a transformation from cellulose I to cellulose II [70] that occurred under the treatment with [C_4_mim]A and the subsequent regeneration of cellulose with water. These results demonstrate that both prepared mILs are able to destruct crystalline parts of cellulose nanofibers, but [C_4_mim]A is much more active in this process. Further investigation of the influence of mILs’ treatment on BC structure and morphology was conducted with an AFM study. The results are given in Figure 5b,c for cellulose regenerated from [C_4_mim]A and ChA, respectively. It is seen that in the case of [C_4_mim]A, the morphology changes from typical for BC interwoven nanofibers to spherical shape granules. At the same time, for ChA, the preservation of a nanofibrous structure is observed. This result is in good agreement with WAXD and DP measurements. Overall, it can be proposed that the imidazolium cation leads to a more pronounced destruction of cellulose including amorphization and depolymerization. The stronger interaction of more polarizable cations with cellulose is in agreement with literature data reported earlier [72,73].

However, it should be taken into account that anions also affect the destruction of cellulose during dissolution. This requires consideration of the impurity of the chloride ions in the obtained acrylates. As noted earlier, a gravimetric analysis of KCl showed that the residual content of the chloride ions in [C_4_mim]A was an order of magnitude lower than ChA. This result is confirmed with the mass spectrometry data given in ESI (Figure S4a and Figure S4b for [C_4_mim]A and ChA, respectively). It can be seen that characteristic patterns for chlorinated compounds connected with the isotopic composition of natural chlorine (^35^Cl and ^37^Cl), namely a series of species with masses M and M + 2, can be noticed for ChA (Figure S4b). At the same time, this is not the case for [C_4_mim]A (Figure S4a). Moreover, literature data indicate that anions containing a carboxylate group may also be active in the dissolution of cellulose, as well as chlorides; for example, 1-ethyl-3-methylimidazolium acetate [74,75]. Both of these factors support the hypothesis that, in the case of synthesized acrylates, the greater activity of [C_4_mim]A in the destruction of the cellulose structure is associated with the structure of the cation.

### 3.6. Rheological Properties of Acrylate-Based mILs with BC

**Figure 6:**
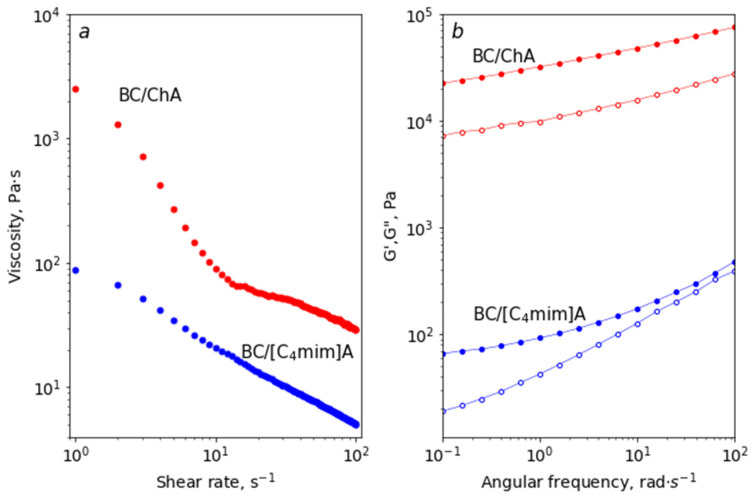
Dependence of the viscosity on the shear rate (**a**); storage (G′, filled symbols) and loss (G″, hollow symbols) moduli as a function of angular frequency (**b**) for BC/ChA and BC/[C_4_mim]A dispersions.

The dependences of viscosity on the shear rate for dispersions containing BC (Figure 6a) demonstrate shear thinning behavior in both cases, which allows the use of such systems for 3D printing [47,76]. The effect of shear thinning is due to the orientation of the BC fibers in the flow direction for the BC/ChA dispersion. In the case of the BC/[C_4_mim]A dispersion, in addition to the orientation of the fibers, spherical particles begin to deform and stretch, as a result of which the viscosity monotonically decreases. 

For the BC/ChA dispersion, higher viscosity values are observed compared to the BC/[C_4_mim]A dispersion (Figure 6a). This correlates with the data on the viscosity of pure mILs and can be attributed to the presence of an –OH group in the choline cation that interacts with the cellulose via hydrogen bonding. In addition, a network of entangled fibers is observed for the BC/ChA dispersion according to the AFM data; although in the case of BC/[C_4_mim]A, the fibers are significantly destroyed (Figure 5b,c). Thus, a greater number of entanglements between the fibers leads to higher viscosity values. It is also worth noting that in the case of BC/[C_4_mim]A, cellulose depolymerization occurs, unlike the system BC/ChA, which can also lead to lower viscosity values of the imidazolium-based mIL.

The results of dynamic frequency sweep tests for the dispersions are shown in Figure 6b. It is seen that the dynamic storage moduli (G′) for both samples surpass the loss moduli (G″) within the whole frequency range, demonstrating the gel-like behavior of the dispersions. Noticeably, the G′ values for BC/ChA (>10^4^ Pa) exceeds BC/[C_4_mim]A by two orders of magnitude, that indicates a higher density of the hydrogen bonding network between cellulose fibers in the choline-based mIL. This may also be due to the presence of an –OH group in the choline cation. Additionally, for the BC/[C_4_mim]A, the G″ is approaching G′ as the dynamic frequency increases to 10^2^ rad∙s^–1^ (loss tangent (tan(*δ*) = G″/G′) becomes close to 1); that is borderline between gel-like and liquid behavior.

An important parameter of the rheological properties of materials for 3D printing is the yield stress—the minimum value of the stress at which the material flows [77]. For the BC/ChA, the yield stress is significantly higher than the yield stress for the BC/[C_4_mim]A, 2030 and 58 Pa (Figure S1b), respectively, which once again, confirms the presence of a stronger structure due to more interlacing between the BC fibers in the case of the BC/ChA dispersion.

**Figure 7:**
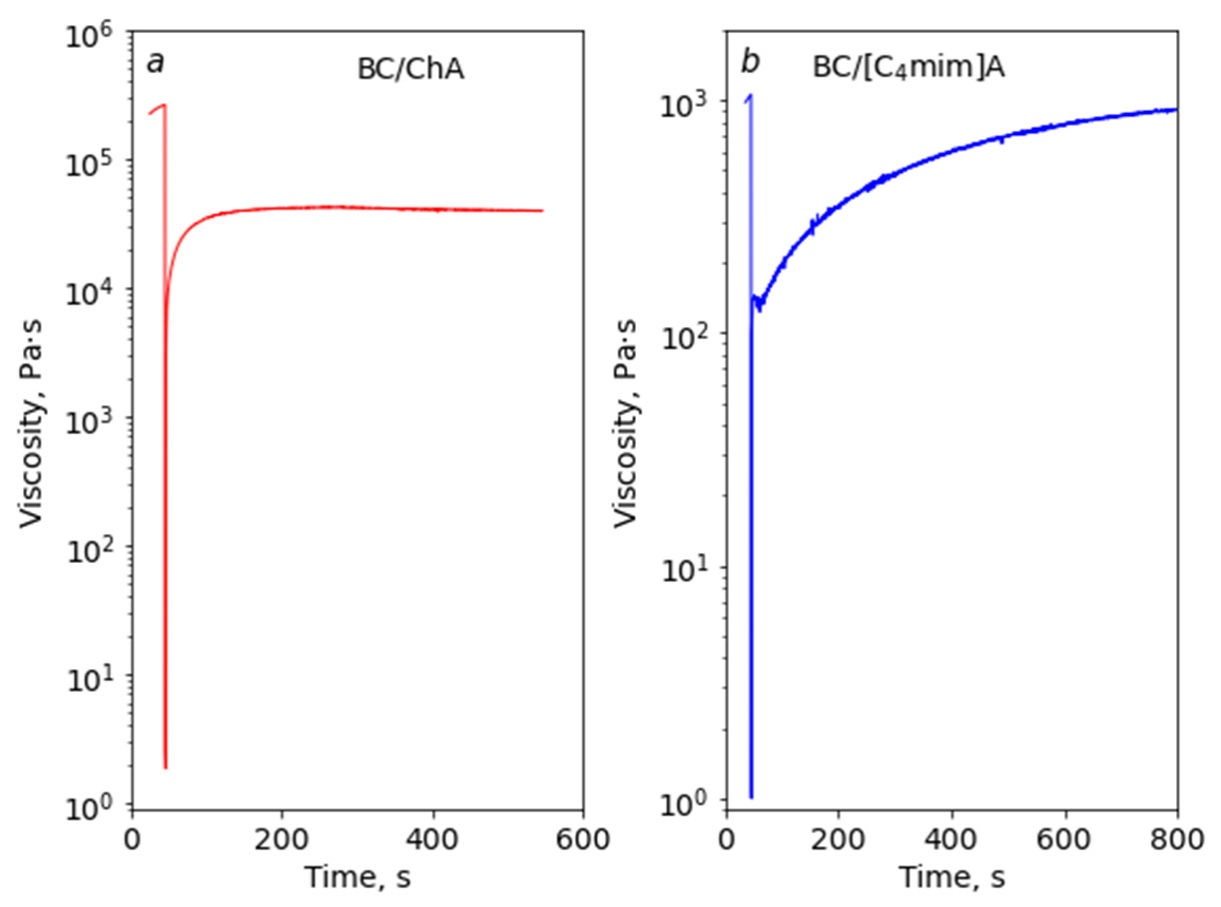
Thixotropy recovery tests for the BC/ChA (**a**) and BC/[C_4_mim]A (**b**) dispersions.

Figure 7 shows the viscosity recovery curves for BC/ChA and BC/[C_4_mim]A after the application of high shear stresses. It can be seen that in the case of the BC/ChA system, the viscosity is restored only to a value of about 2 × 10^4^ Pa∙s, which is lower than the initial value of 2 × 10^5^ Pa∙s. This may be due to significant disentangling and orientation of the BC fibers along the flow, as well as some destruction of fibers.

It is interesting to note that for the cellulose nanocrystals in DES, an almost complete recovery of viscosity values was observed, which suggests that in the case of nanofibers, the process of their reverse relaxation to a tangled state is significantly more inhibited compared to nanocrystals [39]. This result is probably due to the lower mobility of BC nanofibers in dispersion compared to nanocrystals, due to the cooperative effect of the formation of hydrogen bonds by objects with a high aspect ratio.

In the case of the BC/[C_4_mim]A system, the viscosity is restored almost to the initial value, about 10^3^ Pa∙s. This indicates the presence in the dispersion of objects with a decreased aspect ratio, and, consequently, higher mobility compared to nanofibers that are present in the BC/ChA system.

### 3.7. 3D Printing and Mechanical Properties

The production of functional cellulose-based gels by additive technologies (3D printing) is intended to expand their application areas. For example, composite gels containing cellulose nanofibers with a stable crystalline structure and morphology have been used for the reinforcement of 3D printed scaffolds [78] and medical-grade resin [79]. At the same time, inks with fully or partially dissolved cellulose in ILs have been employed for 3D bioprinting [80,81], the production of gel-electrolytes in flexible energy storage systems [82,83], mechanical sensors (e-skin) [84], and stiffness-changing smart materials [85].

**Figure 8:**
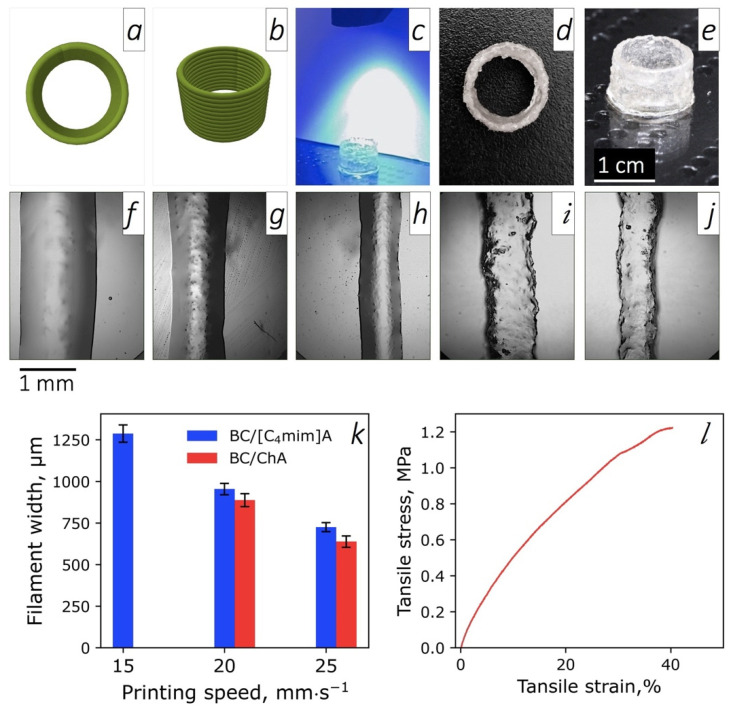
The CAD model of the hollow cylinder (**a**,**b**); photos of the photocuring process at 3D printing (**c**); and the resulting cylinder in various views (**d**,**e**). The optical micrographs of 3D printed filaments (magnification of ×10) for BC/[C_4_mim]A ink (**f**–**h**); at the printing pressure of 50 kPa and BC/ChA ink (**i**,**j**); at the pressure of 250 kPa at varied nozzle moving speeds of 15 (**f**), 20 (**g**,**i**), and 25 mm·s^–1^ (**h**,**j**). The dependences of filament width on the printing speed (**k**). Typical stress-strain curve for the UV-cured BC/ChA sample (**l**).

To characterize the applicability of prepared inks for 3D printing of complex shaped objects, a CAD model of a hollow cylinder was designed and sliced using GeSiM Robotics software (Figure 8a,b). During the printing, each layer was UV-cured with an intensity of 2 W∙cm^–2^ for 10 sec (Figure 8c). The photos of a 3D printed cylinder based on BC/ChA ink with a diameter of 12 mm and a height of 10 mm are presented in Figure 8d,e. At the same time, due to a lower viscosity of BC/[C_4_mim]A ink, the 3D printing of a cylinder was complicated because of considerable filament spreading during the stacking of the layers. To evaluate the 3D printing accuracy for the prepared inks, several filament lines were printed on a glass substrate at a variable nozzle moving speed. As is seen from the optical micrographs presented in Figure 8f–h, the surface of the filaments based on BC/[C_4_mim]A ink is smooth due to the partial dissolution of BC, while the surface of the BC/ChA filaments is scabrous (see Figure 8i,j). One can assume that a strong spatial network of cellulose fibers and their possible partial agglomeration in BC/ChA ink leads to a non-uniform flow at extrusion. It is worth noting that the required plunger pressure for 3D printing of BC/ChA ink was five times higher compared with BC/[C_4_mim]A ink. This is in agreement with results of rheological measurements demonstrating that the apparent viscosity of BC/ChA ink at the high shear rate (10^2^ s^–1^) exceeds one for BC/[C_4_mim]A ink: 29.3 Pa·s vs. 5.1 Pa·s, respectively. Due to the higher viscosity and rapid thixotropic recovery rate of BC/ChA ink, a higher resolution of 3D printing can be achieved. As shown in Figure 8k, the BC/ChA-based filaments have a thickness of 628 ± 34 μm, while the BC/[C_4_mim]A sample demonstrate 725 ± 27 μm at the same printing speed of 25 mm·s^–1^ and an outlet diameter of the printing nozzle of 580 μm.

Mechanical properties of UV-cured gels were investigated in the uniaxial tension mode. The samples based on the BC/[C_4_mim]A dispersion were too weak for mechanical testing, that was not the case for the BC/ChA one. Nevertheless, the BC/[C_4_mim]A samples could be easily detached from the substrate, maintaining their integrity under their own weight and demonstrating mechanical flexibility that may be sufficient for some applications. Interestingly, the DES based on a mixture of [C_4_mim]Cl and AA in the presence of BC (1 wt%) was UV-polymerized, resulting in well-defined mechanical characteristics [47]. The reasons for the diminished mechanical properties of BC/[C_4_mim]A can be associated with two factors: depolymerization of the cellulose in [C_4_mim]A and the destruction of the morphology of the BC, which leads to a decrease in the number of intermolecular hydrogen bonds in cellulose.

The typical stress-strain curve for the BC/ChA sample is shown in Figure 8l. The Young’s modulus (*E*) for the BC/ChA gel sample is 7.1 ± 1.0 MPa, while the fracture stress is σ = 1.2 ± 0.1 MPa and the elongation at the break is ε_b_ = 40 ± 5%. The gel obtained from the BC/ChA ink was mechanically stronger than ones containing ChCl reinforced with cellulose. For example, gels based on polymerized DES (PDES) based on AA and ChCl containing 1 wt% of BC have demonstrated σ = 0.56 MPa and *E* = 0.56 MPa [47]. PDES (AA/ChCl) synthesized in BC pellicle demonstrated σ = 0.8 MPa at ε_b_ = 290% [86]. The obtained elastic modulus are also higher than one for [C_4_mim]Cl entrapped in a poly(1-vinylimidazole-co-AA) matrix demonstrating σ = 1.6 MPa at ε_b_ = 1090% [87] and [C_4_mim](trifluoromethylsulfonyl)imide in a poly(ethylene glycol methacrylate-co-N-vinyl-3-butyl imidazole bis(trifluoromethylsulfonyl)imide) matrix with σ up to 0.3 MPa at ε_b_ = 16.5% [88].

## 4. Conclusions

The possibility of the preparation of polymerizable 1-butyl-3-methylimidazolium and choline acrylates ([C_4_mim]A and ChA, respectively) using the modified Fukumoto method from corresponding chlorides was successfully demonstrated. The FTIR and NMR results confirm the chemical structure of the prepared monomeric ionic liquids (mILs). Both acrylates demonstrate the typical for mILs glass transition temperature of around 180 K and a melting point of about 310 K. The gradual decreasing symmetry of the chemical surroundings of the carboxylate groups was found in the temperature range between *T*_g_ and *T*_m_ from the FTIR results. The viscosity of ChA was higher than [C_4_mim]A, which is explained by the hydrogen bonding between the –OH group of choline cations and the –COO^−^ group of acrylates. The prepared mILs were able to form stable dispersions of bacterial cellulose (BC). The destruction of the BC morphology and crystalline structure, as well as depolymerization, was observed in [C_4_mim]A. At the same time, using ChA resulted in the stability of the cellulose structure. 

The partial recovery of initial viscosity of the BC dispersions in the mILs was found in both inks. However, the dispersion based on ChA demonstrated higher viscosity in comparison with the ones based on [C_4_mim]A by 1.5–2 orders of magnitude. Faster thixotropic response also makes the BC/ChA dispersion more suitable for 3D printing. The difference in rheological characteristics results in higher shape fidelity for the BC/ChA ink in comparison with the BC/[C_4_mim]A one. The applicability of the BC dispersions in prepared mILs for 3D printing with subsequent UV-curing in the presence of N,N′-methylenebisacrylamide as a crosslinker was demonstrated. Measurements of the mechanical properties revealed that the curing process was more successful in the case of ChA in comparison with [C_4_mim]A: the mechanical properties of polymerized BC/[C_4_mim]A was not possible to measure, while the polymerized BC/ChA demonstrated average values of Young’s modulus of 7.1 ± 1.0 MPa with 1.2 ± 0.1 MPa and 40 ± 5% of strength and ultimate elongation, respectively. The obtained values are higher than the ones reported in the literature for similar ionic liquid/carbohydrate polymer materials. Higher mechanical characteristics of the BC/ChA material can be explained by the preservation of cellulose DP in ChA in comparison with the [C_4_mim]A, in which the destruction of cellulose fiber morphology was observed. All these results demonstrate the benefit of the study of UV-curable inks based on choline cations for the preparation of cellulose-based inks for 3D printing over imidazolium-based ones.

## Figures and Tables

**Table 1:** Degree of polymerization (DP) of the initial BC and after regeneration from mILs.

Sample	DP
Initial BC	730
BC after ChA	715
BC after [C_4_mim]A	370

## Data Availability

Not applicable.

## Supplementary Materials

The following are available online at https://www.mdpi.com/article/10.3390/polym14235148/s1, Figure S1: Storage (G′, filled symbols) and loss (G″, hollow symbols) moduli as a function of strain (a) and shear stress (b) for BC/ChA and BC/[C_4_mim]A dispersions. Figure S2: ^1^H NMR spectra of the obtained monomeric ionic liquids: [C_4_mim]A (a) and ChA (b); D_2_O was used as a solvent. Figure S3: ^13^C NMR spectra of the obtained mILs: [C_4_mim]A (a) and ChA (b); D_2_O was used as a solvent. Figure S4: Electrospray mass spectra of [C_4_mim]A (a) and ChA (b) in negative mode.
