# Supplementary material for: Synthesis and Physicochemical Properties of Acrylate Anion Based Ionic Liquids
Source: Polymers (Basel). 2022 Nov 26;14(23):5148. doi: 10.3390/polym14235148 (PMC9736722; doi:10.3390/polym14235148)
Supplement: Supplementary file 1 [file polymers-14-05148-s001.zip › polymers-1968444-supplementary.pdf]

Polymers

ELECTRONIC SUPPLEMENTARY INFORMATION FOR

Synthesis and physicochemical properties of acrylate anion based ionic liquids

Veronika S. Fedotova[1], Maria P. Sokolova[1,*], Vitaliy K. Vorobiov[1], Eugene V. Sivtsov [2], Mauro C. C. Ribeiro[3], Michael A. Smirnov[1,4,*]

[1] Institute of Macromolecular Compounds, Russian Academy of Sciences, V.O. Bolshoi Pr. 31, 199004 St. Petersburg, Russia

[2] Saint Petersburg State Institute of Technology, Moskovsky prospekt 24-26/49, 190013 St. Petersburg, Russia

[3] Laboratório de Espectroscopia Molecular, Departamento de Química Fundamental, Instituto de Química, Universidade de São Paulo, 05513-970 São Paulo, Brazil

[4] Institute of Chemistry, Saint Petersburg State University, Universitetsky pr. 26, Peterhof, 198504 St. Petersburg.

* Correspondence: pmarip@mail.ru (M.P.S.); smirnov_michael@mail.ru (M.A.S.)

1. Rheological properties of acrylate-based mILs with BC

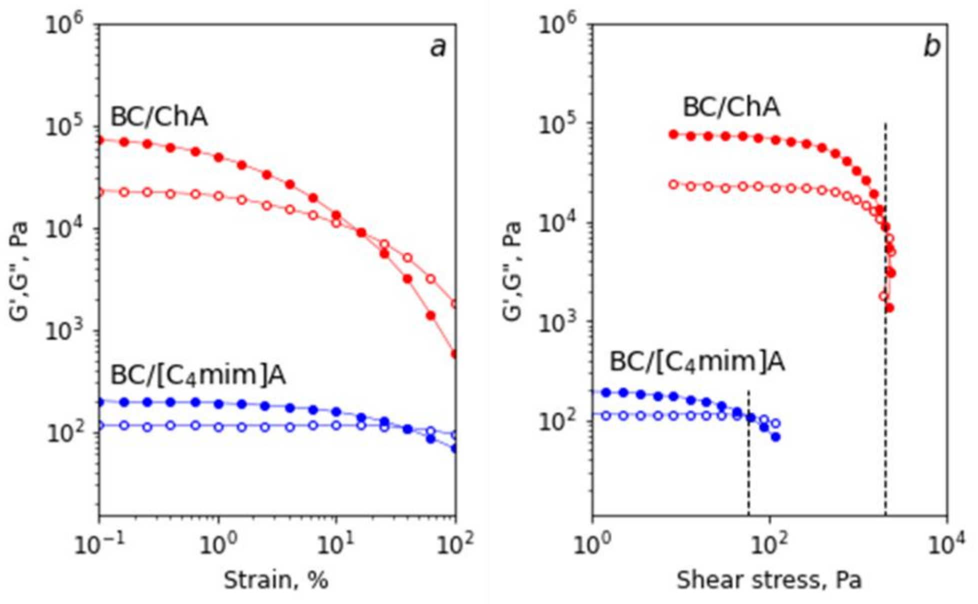

Figure S1. Storage (G′, filled symbols) and loss (G″, hollow symbols) moduli as a function of strain (**a**) and shear stress (**b**) for BC/ChA and BC/[C_4mim]A dispersions.

2. Structural study of mILs by ^{1}H NMR spectroscopy

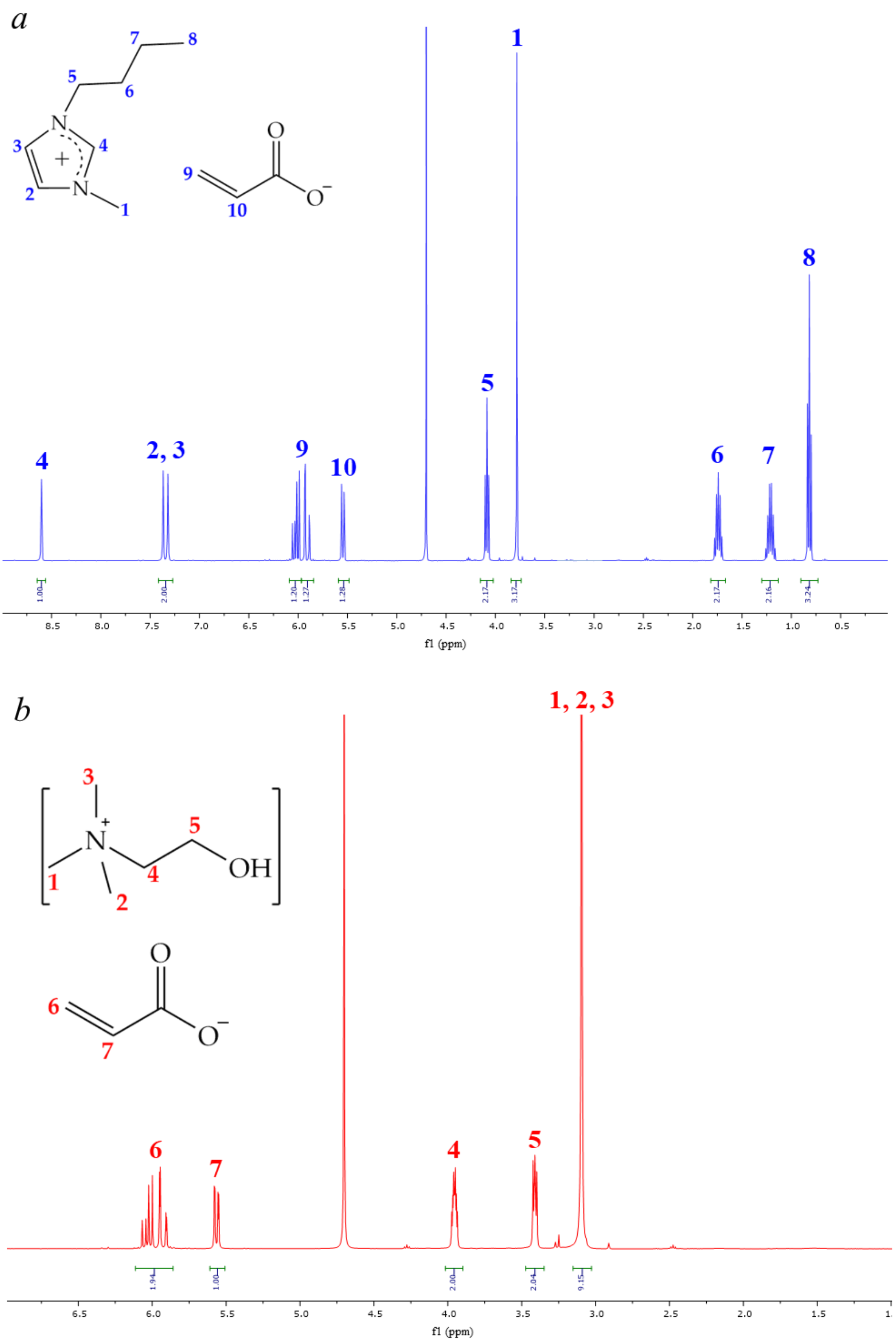

Figure S2. ^{1}H NMR spectra of the obtained mILs: $[C_4mim]A$ (**a**) and ChA (**b**); D_2O was used as a solvent.

3. Structural study of mILs by ^{13}C NMR spectroscopy

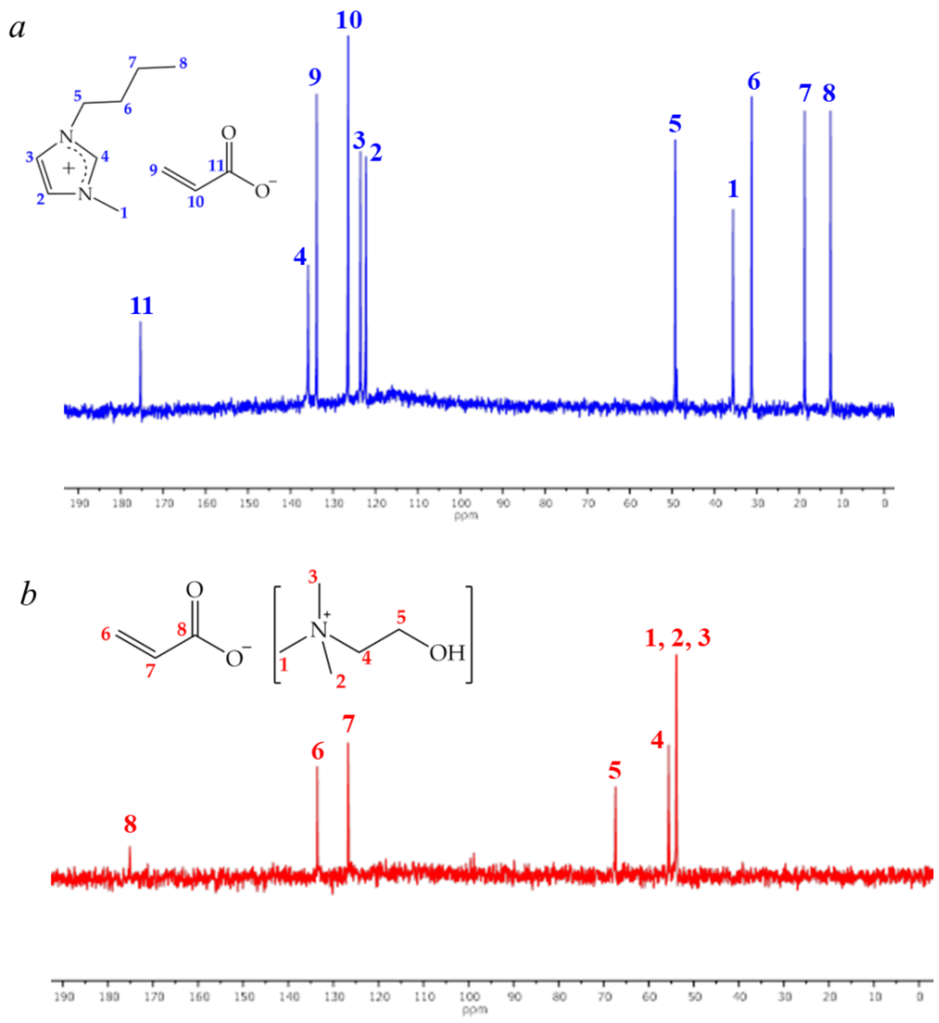

Figure S3. ^{13}C NMR spectra of the obtained mILs: [C_4mim]A (**a**) and ChA (**b**); D_2O was used as a solvent.

4. Study of mILs by mass spectrometry

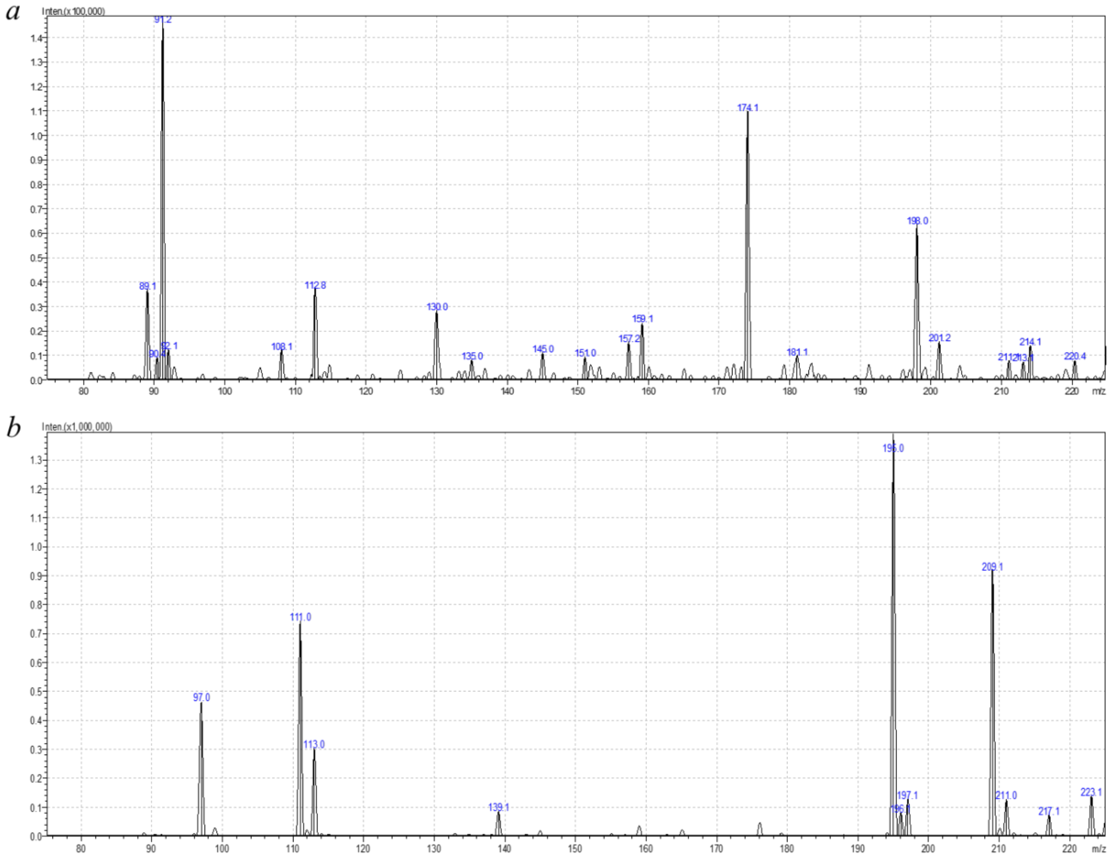

Figure S4. Electrospray mass spectra of [C_4mim]A (**a**) and ChA (**b**) in negative mode.
